# 간호대학생 대상의 고충실도 시뮬레이터를 이용한 분만 간호 교육프로그램의 개발 및 효과

**DOI:** 10.4069/kjwhn.2020.09.18

**Published:** 2020-09-29

**Authors:** Seo-A Park, Hye Young Kim

**Affiliations:** 1 Department of Nursing, Graduate School, Keimyung University, Daegu, Korea; 1 계명대학교 대학원 간호학과; 2 College of Nursing, Keimyung University, Daegu, Korea; 2 계명대학교 간호대학

**Keywords:** Education, Maternal-child nursing, Students, Simulation training, 교육, 모성간호, 대학생, 시뮬레이션 교육

## Abstract

**Purpose:**

This study was conducted to investigate the effects of an education program using a high-fidelity simulator of labor and delivery on nursing knowledge, critical thinking, and clinical performance among nursing students who had not yet experienced clinical practicum.

**Methods:**

The development of a 5-week maternity nursing education programs using high-fidelity simulators included modules containing case-oriented scenarios, knowledge, and skills required for maternity care. A randomized controlled study was conducted to verify the effects of the developed program. Data were collected from October 21 to December 9, 2019. The experimental group (n=36) participated in a 5-week high-fidelity simulation program on care for the woman in labor, whereas the control group (n=36) received standard education as lecture and practice with delivery model. The collected data were analyzed using descriptive statistics (frequency, percentage, mean, and standard deviation), the Chi-square test, Fisher exact test, and t-test.

**Results:**

For participants who received education using the high-fidelity simulation program, nursing knowledge (t=2.33, *p*=.011), critical thinking (t=3.73, *p*<.001), and clinical performance (t=2.53, *p*=.006) were significantly higher than in the control group.

**Conclusion:**

Even for students with no clinical experience, high-fidelity simulation-based nursing education was effective in improving nursing knowledge, critical thinking, and clinical performance among nursing students. Nurse educators will be able to use this high-fidelity simulator effectively, especially in situations where direct clinical practicum may not be feasible.

## Introduction

### 연구 필요성

간호대학생은 간호교육을 통해 필요한 지식과 기술을 습득하여 전문직 능력을 갖춘 간호사로 성장한다. 간호교육은 크게 이론수업과 임상실습으로 구분되며, 이 중 임상실습교육은 이론수업에서 배운 내용을 실제 임상현장에 적용하는 교육과정이다[1]. 그러나 현재 한정된 임상실습현장에 비해 간호교육 기관과 간호대학생의 수는 증가하고 있어 간호대학생이 환자를 직접 간호할 수 있는 기회가 줄어들고 있다[2,3]. 이와 같이 간호대학생의 관찰 위주 실습교육 증가로 인해 술기수행의 기회가 감소되고 학교에서 배운 지식을 적용하기 어려워지고 있다[4].

최근 저출산 문제로 2017년에는 합계 출산율이 역대 최저 수준인 1.05명까지 하락했으며, 출생아 수 또한 공식 통계 작성 이래 처음으로 40만 명 아래로 떨어졌다고 보고하였다[5]. 임산부들은 자신의 출산 경험을 의료인이 아닌 실습 학생들과 공유하는 것을 꺼려하고 있어, 임상실습교육에서 간호대학생들이 자연분만 과정을 경험하기는 어려운 실정이다[2,4]. 이러한 상황에서 전통적인 교수법만으로는 간호대학생들이 분만 과정에서 필요로 하는 간호학적 역량과 문제해결능력을 충족하지 못하고 있어, 대안이 시급한 실정이다[2,6,7]. 이러한 임상실습교육의 한계점을 극복하기 위하여 임상현장과 유사한 상황을 구현할 수 있는 시뮬레이션 교육이 대두되어 현장실습을 보완하는 한 형태로 자리잡게 되었다. 시뮬레이션 교육은 임상의 실제 상황과 비슷한 시나리오를 사용하여 안전한 환경에서 핵심 술기능력, 대상자 및 의료인과의 의사소통능력, 간호수행능력, 임상수행능력, 비판적 사고능력, 환자 안전관리능력, 자신감 향상 등을 습득할 수 있는 장점이 있다[2,8,9].

산과 영역에서는 국내외 조산사 및 전공의를 대상으로 분만 시뮬레이터를 이용해 제왕절개, 흡인분만, 난산, 고위험 분만 관리 등과 같이 임상현장에서 접하기 어려운 상황에 대해 시뮬레이션 실습교육을 실시함으로써 긍정적인 교육의 효과를 거두고 있다고 보고하였다[10-12]. 그러나 현재 산과 간호 영역에서의 시뮬레이션 실습교육에 관한 연구로는 술기수행능력[6,13], 교육 만족도[6,7], 수행 자신감[13-15] 등 지식과 기술에 초점을 맞춘 제한점이 있고, 비판적 사고성향과 임상 판단력, 문제해결능력 등 역량과 관련 있는 변수를 측정한 연구는 드문 실정이다. 특히 분만실과 산과병동에서 간호사의 비판적 사고능력과 임상 판단에 따른 수행능력은 매우 중요하다[7]. 과거 분만과 관련된 업무만을 담당하던 분만실은 현재 임신부터 출산에 이르는 임산부의 전반적인 관리 및 산과 수술실의 기능도 수행하고 있다[16]. 또한 최근 고위험 산모의 증가와 함께 분만실 간호사의 역할과 책임은 더욱 증가되고 있으며, 분만실 간호사의 역량 개발과 전문직 교육이 강조되고 있다[17]. 따라서 이러한 임상현장의 요구를 해결하기 위하여 간호대학생들은 일정 수준 이상의 임상수행능력을 갖춘 신규 간호사로 기본적인 역량을 갖춰야만 한다[13,18]. 현재 제한된 임상실습 환경으로 인해[2] 대부분의 간호대학생들은 분만실 실습 시 분만 간호를 총체적으로 경험하지 못하고 분만의 단계를 부분적으로 경험하고 있다. 그러므로 간호대학생들은 시뮬레이션 기반 학습을 통해 통합적, 비판적 사고에 기반한 총체적인 접근을 할 수 있는 임상수행능력을 함양할 필요가 있다[18].

고충실도 시뮬레이션 교육프로그램은 쌍방향 학습이 가능하며, 간호대학생의 비판적 사고성향과 임상 판단력, 임상수행능력, 문제해결능력 등을 향상시킨다고 보고하고 있다[19,20]. 고충실도 시뮬레이션 교육프로그램은 임상과 유사한 환경을 재현함으로써 학생들이 자신감, 만족도, 환자에 대한 불안 감소 등의 긍정적 경험을 갖게 한다[20]. 이에 본 연구는 간호대학생을 대상으로 고충실도 시뮬레이터를 이용하여 간호 지식, 비판적 사고성향 및 임상수행능력을 획득할 수 있는 프로그램을 개발하고 실제와 유사한 임상 환경의 상황을 사례로 적용하여 분만 시뮬레이션 교육의 효과를 검증하고자 하였다. 또한 고충실도 시뮬레이터를 이용한 시뮬레이션 교육의 효과를 파악하여 더 체계적이고 실질적으로 유용한 시뮬레이션 실습 교과과정 개발의 기초자료를 마련하고자 시도하였다.

### 연구 목적 및 가설

본 연구의 목적은 고충실도 시뮬레이터를 이용한 분만 간호 교육프로그램을 개발하여 임상실습교육을 앞둔 3학년 간호대학생을 대상으로 분만 간호 지식, 비판적 사고성향, 임상수행능력에 미치는 효과를 파악하기 위함이다.

1) 고충실도 시뮬레이터를 이용한 분만 간호 교육프로그램을 적용한 실험군은 대조군보다 분만 간호 지식 점수가 높을 것이다.

2) 고충실도 시뮬레이터를 이용한 분만 간호 교육프로그램을 적용한 실험군은 대조군보다 비판적 사고성향 점수가 높을 것이다.

3) 고충실도 시뮬레이터를 이용한 분만 간호 교육프로그램을 적용한 실험군은 대조군보다 임상수행능력 점수가 높을 것이다.

## Methods

Ethics statement: This study was approved by the Institutional Review Board of Keimyung University (40525-201909-HR-054-02). Informed consent was obtained from the participants.

### 연구 설계

**Figure. 1.**
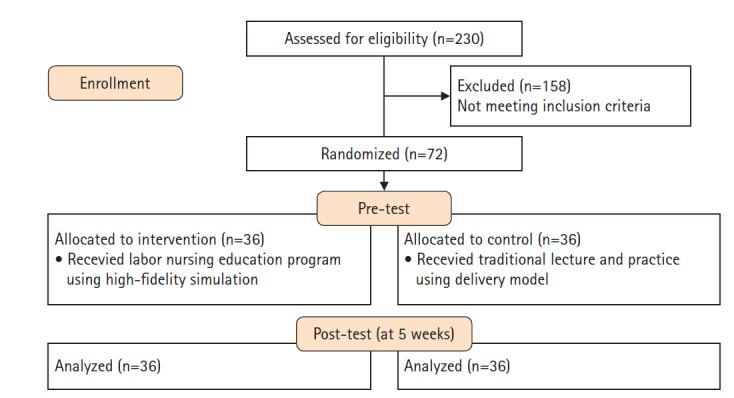
Flow diagram of participants.

본 연구는 간호대학생 대상의 고충실도 시뮬레이터를 이용한 분만 간호 교육프로그램이 분만 간호 지식, 비판적 사고성향, 임상수행능력에 미치는 효과를 분석하기 위해 무작위 대조군 실험설계를 이용하였다(Fig. 1).

### 연구 대상

본 연구의 대상은 경북 김천시에 소재한 대학 3학년 간호대학생으로 연구 목적을 이해하고 자발적으로 연구에 참여한 대상자로 선정하였다. 구체적 선정기준으로는, 3학년 1학기 모성간호학 교과목을 이수하였으며 시뮬레이션 실습 경험 및 산부 간호에 대한 임상실습 경험이 없는 자로 하였다. 연구 대상자의 표본 수는 간호대학생을 대상으로 시뮬레이션 교육을 실시한 Kim과 Ha [7]의 비판적 사고성향에 대한 효과크기가 0.66으로 확인되었다. 따라서 G*power ver. 3.1.0 프로그램을 활용하여 군의 표본의 수의 크기에 필요한 효과크기는 유의수준(α) .05, 검정력 (1–β) .80, 효과의 크기(d) .66를 기준으로 두 집단의 평균 비교에 필요한 표본의 수를 산출한 결과 총 60명으로 실험군과 대조군 각 30명이었으며, 탈락률을 20%로 고려하여 각 36명으로 자료를 산출하였다. 연구 진행 중 두 집단에서 탈락자는 없이 총 72명으로 최종 자료를 수집하였다. 중재 오염의 위험을 줄이기 위해 밀봉된 봉투에 일련번호를 매긴 후 짝수는 실험군으로, 홀수는 대조군의 무작위 할당하였다. 또한 지원한 대상자들을 분반하여 진행하였으며, 실험이 오염될 수 있는 가능성을 최소화하기 위하여 실험군과 대조군의 층을 달리하여 중재를 제공하였다.

### 연구 도구

#### 분만 간호 지식

분만 간호 지식은 모성간호학 학습목표 및 문헌을 바탕으로 분만 간호의 핵심적인 지식에 대해 연구자가 개발한 문항으로 측정하였다. 모성간호학 전공 교수 3인, 10년 이상의 분만실 경험을 가진 현직 간호사 1명, 5년 이상 산부인과 병동 경험을 가진 현직 간호사 2명에게 내용타당도를 검증받았다. 내용타당도 검증 시 수정이 필요한 부분에 대한 전문가 의견을 기술하도록 하여 수정, 보완하였으며 항목별 내용타당도는 0.8–1.0이었다. 최종적으로 개발된 도구는 분만 간호에 대한 15문항으로 구성하였다. 각 문항에 대해 정답은 1점, 오답과 잘 모른다는 0점으로 처리하였고 총 점수의 범위는 0–15점으로 점수가 높을수록 분만 간호 지식 점수가 높은 것을 의미한다. 본 연구에서 도구의 신뢰도 Cronbach’s α는 .81이었다.

#### 비판적 사고성향

비판적 사고성향의 측정은 Yoon [21]이 간호대학생을 대상으로 문제해결과 의사결정을 이끌어 내기 위해 개발한 평가도구를 저자의 허락 하에 사용하였다. 본 도구는 지적 열정/호기심 5문항, 신중성 4문항, 자신감 4문항, 체계성 3문항, 지적 공정성 4문항, 건전한 회의성 4문항, 객관성 3문항의 7개 요인 27개 문항으로 구성되어 있다. 각 문항은 ‘전혀 그렇지 않다’ 1점부터 ‘매우 그렇다’ 5점의 Likert 척도로 총 점수의 범위는 27–135점까지이며, 점수가 높을수록 비판적 사고성향이 높음을 의미한다. Yoon [21]의 연구에서 Cronbach’s α는 .84이었고, 본 연구에서 도구의 신뢰도 Cronbach’s α는 .79였다.

#### 임상수행능력

임상수행능력은 Lee 등[22]이 간호학생을 대상으로 개발한 임상수행능력 도구를 Choi [23]가 수정, 보완한 도구를 저자의 허락 하에 사용하였다. 이 도구는 임상수행능력에 관한 5가지 영역을 측정하는 도구로 전문직 발전 9문항, 간호기술 11문항, 간호교육/협력관계 8문항, 대인관계/의사소통 6문항, 간호과정 11문항의 총 45문항으로 구성되어 있다. 각 문항은 ‘매우 못한다’ 1점부터 ‘매우 잘 한다’ 5점의 Likert 척도로 총 점수의 범위는 45–225점까지이며, 점수가 높을수록 임상수행능력이 높음을 의미한다. Lee 등[22]이 개발한 전체 문항 신뢰도 Cronbach’s α는 .96, Choi [23]의 연구에서 Cronbach’s α는 .92였으며, 본 연구에서 도구의 신뢰도 Cronbach’s α는 .95였다.

### 고충실도 시뮬레이터를 이용한 분만 간호 교육프로그램 개발

고충실도 시뮬레이터를 이용한 분만 간호 교육을 시행하기 위해 산부 간호에 요구되는 핵심 간호기술과 중요한 간호문제를 중심으로 한 모듈을 개발하였다(Table 1). 모듈은 분만 진행 중에 일어날 수 있는 상황으로 진통을 겪는 대상자의 건강 문제를 사정하고 간호를 계획하며 이에 적합한 중재에 필요한 지식, 심리, 정서적 간호를 포함한 술기 및 태도 등을 포함하였다. 또한 평가항목 역시 이러한 요소를 바탕으로 구성하였다.

본 연구의 프로그램 설계 단계에서는 간호학 교수 4인으로 구성된 전문가 회의를 두 차례 진행하였다. 1차 회의에서는 교육의 목표와 교육 방법, 성취도 평가 방법, 주차별 내용에 대한 논의를 진행하였고, 2차 회의에서는 구체적인 주제 및 내용을 선정하였다. 산부 간호 시뮬레이션 교육프로그램의 주제 및 내용 선정을 위해 대한간호협회에서 제시한 모성간호학 학습목표[24] 및 주 교과서[25]를 토대로 학습내용을 구성하였다. 산부 간호의 구체적인 교육내용은 자궁수축 및 분만 진행과정 사정, 자궁 저부 높이 측정, 무자극 검사의 적용 및 태아 전자감시 결과 해석, 레오폴드 촉진법, 분만 중 통증 완화, 호흡법, 분만 중 대상자와 가족을 위한 심리적 간호 등 분만에 따른 포괄적인 간호를 포함하였다. 이를 토대로 산부 간호의 학습목표와 학습내용을 포함한 임상사례 기반의 시나리오를 작성하여 시뮬레이션 모듈을 개발하였다. 시뮬레이션 시나리오 선정 및 임상수행 체크리스트 구성은 모성간호학 담당 교수 2인과 분만실 경력 10년차 간호사 1인의 자문을 받아 진행되었다. 주제와 관련하여 대상자로부터 정보 수집 및 신체검진, 관련 임상병리검사 결과 해석, 수집된 자료 분석 및 간호계획 수립, 간호중재 수행, 의사소통, 관련된 간호술기 연습을 다룰 수 있도록 모듈을 구성하였다.

고충실도 분만 시뮬레이션 간호 교육프로그램에 참여하는 학생과 교육자를 위해 학생용 지침서와 교육자용 지침서를 제작하였다. 지침서는 프로그램 설계에 참여했던 교수 4인의 검토를 거쳐 제작되었으며, 회의를 통해 내용 점검 후 확정하고 배포하였다. 지침서는 교육자와 학생이 숙지해야 하는 내용 및 프로그램 개요, 사전 교육내용, 성찰일지 등으로 구성되었다.

### 연구 진행 절차

2019년 10월 21일부터 2019년 12월 09일까지 김천시에 소재한 대학 3학년 간호대학생으로 하였다. 대상자 모집은 간호대학생을 대상으로 연구홍보물을 게시하고 참여관심을 보인 학생에게는 연구자가 연구의 목적, 필요성, 방법 등에 대해 설명하였으며, 본 연구 참여는 정규교육과정에 포함되지 않는다는 것을 공지한 후 자발적으로 참여 의사를 보인 대상자에게 서면동의서를 받았다. 대상자 개인정보의 비밀보장, 익명성을 보장하며, 수집된 자료는 연구목적 이외에 사용되지 않음을 설명하였다. 연구 중 언제라도 참여를 철회가 가능함을 설명하였다. 자료수집은 중재 오염의 위험을 줄이기 위하여 지원한 대상자들을 분반하여 진행하였으며 실험군과 대조군의 층을 달리하여 중재를 제공하였다. 본 연구의 참여를 독려시키기 위하여 대상자들에게 교육 전 SNS 및 전화를 활용하여 매회 교육 참여 여부를 재 확인하였으며, 설문조사가 완료된 후에는 감사의 표시로 소정의 상품을 제공하였다.

#### 사전 조사

사전 조사는 두 군 모두 중재 1일째에 일반적 특성, 지식, 비판적 사고성향, 임상수행능력에 대해 설문지로 조사하였으며, 작성시간은 20분 정도 소요되었다(Table 1).

#### 중재 제공

개발된 고충실도 시뮬레이터를 이용한 분만 간호 교육프로그램의 운영은 교과 과정의 특성, 시뮬레이션 실습실 환경과 실습 학생 수를 고려하여 구성하였다. 시뮬레이션 실습교육 운영은 주당 1회 2시간씩 5차의 고충실도 시뮬레이션 기반 교육을 개별 및 조별 학습으로 진행하였다. 조별 학습은 학생들의 참여와 학습효과를 증진하기 위해 5명 이하의 소집단 활동이 권장되므로[26], 실습팀 당 학생 수는 4명으로 하였다. 시뮬레이션 교육은 동일한 환경을 재현한 두 곳에서 이루어지며, 교수자 1인은 시뮬레이션 교육 운영을 담당하고 평가하고, 연구 보조원 1인은 전체 시뮬레이션 실습교육 운영을 도왔다. 연구 보조원 1인은 임상 경력 10년 이상인 간호사로 실습 조교로 근무 중이다. 연구 보조원 훈련을 위해 연구의 목적과 절차 및 시나리오에 대한 내용을 설명하고 연구자와 평가자 간 논의를 통해 시나리오에 맞는 구체적 간호내용을 평가할 수 있도록 항목별로 확인하면서 평가방법을 훈련하였다. 시뮬레이션 실습교육은 학습목표 제시와 함께 주제와 관련된 사전학습, 관련 간호실습, 시나리오 경험, 디브리핑(debriefing) 순으로 시행하였다. 디브리핑은 비디오 녹화 후 조별로 확인하여 수행한 간호의 적절성 및 시뮬레이션 교육에 대해 느낀 점을 자유롭게 표현하도록 하였다. 교수자는 학생들의 간호수행에 따른 평가 후 질의응답 시간을 가졌다. 교육매체는 PowerPoint (Microsoft, Redmond, WA, USA)와 교재를 활용하여 중재를 제공하였으며, 사전에 제작된 지침서를 통하여 학생들의 시뮬레이션 실습 활동을 기록하도록 하였다. 고충실도 시뮬레이터를 이용한 분만 간호 교육프로그램의 효과 검증은 프로그램 참여 전에 간호 지식, 비판적 사고성향, 임상수행능력에 대한 사전 조사를 시행하고 프로그램 종료 시점에 사후 조사를 시행하였다(Table 1).

• 실험군: 본 연구의 실험군의 교육프로그램 중재 방법으로 고충실도 시뮬레이터를 이용한 분만 간호 중재를 제공하였다. 본 연구에서 실시한 시뮬레이션 교육은 Laerdal사(Stavanger, Norway)에서 만든 고충실도 시뮬레이터인 SimMom Laerdal을 활용하였다. 실제 임상현장의 상황을 사례로 적용하였으며, 고충실도 시뮬레이터 SimMom을 산부로 가정하고 분만 교육을 진행하였다.

교육프로그램 1주차 세션은 자궁 수축, 분만 요소, 분만 기전, 통증 및 불편감 사정, 태아 전자감시기에 따른 산부 간호 교육 및 핵심 이론을 설명, 시뮬레이션 실습 진행 방법에 대한 소개를 2시간 실시하였다. 2–3주차 세션은 고충실도 시뮬레이터를 이용하여 분만 간호 중재를 훈련하는 단계로, 36명이 4명씩 한 조를 이루어 총 9조로 구성되어 실습에 참여하였다. 교수자는 시뮬레이션 실습실의 구조 및 환경 설명과 고충실도 시뮬레이터의 기능, 물품 사용 등 수업진행 절차에 대해 설명한 후 사례 기반 분만 시나리오를 제공하였다. 고충실도 시뮬레이션 교육을 위하여 시나리오는 규칙적인 진통이 있는 임신 39주 2일 된 초산부(32세)의 상황으로 선정하였다. 구체적인 시나리오 상황은 다음과 같다. 임신 39주 2일이 된 초산부는 내원 5시간 전부터 규칙적인 진통과 혈성 이슬이 관찰되어 산부인과 외래를 통해 분만실로 입원하였다. 분만실로 입원한 산부는 아직 자궁 수축과 태아 심음 확인을 위한 모니터는 부착하지 않은 상태이며, 의사의 처방에 따라 혈액검사 및 자궁 수축 상태와 태아 심음을 확인하여야 한다. 대상자는 진통이 올 때마다 얼굴을 찡그리며 아기가 괜찮은지 궁금해하며, 주 증상과 관련하여 가족은 불안해하는 상황이다. 학생들에게 대상자의 이름, 나이, 키, 체중, 과거력, 산과력, 가족력, 검사 결과, 약물, 처치 내용, 임상병리검사 및 환자 진단 등의 환자 정보를 제시하였다. SimMom은 고충실도 시뮬레이터로 임신 39주 2일이 된 초산부로 가정하고, 학생들은 분만실 간호사의 역할을 하게 된다. 학생들은 분만실 간호사로서 상황의 진행에 따라 레오폴드 복부 촉진법 시행, 태아 전자감시기 적용 및 결과 해석, 태아 사정, 의식 및 신체 사정, 산소요법, 호흡법, oxytocin 약물 사용법, 의사소통, 심리적 불안 완화, 산부 및 가족의 정서적 지지 등의 간호를 계획하고 수행하도록 하였다. 조별로 시뮬레이션 실습을 진행하였는데, 실험군의 모든 학생은 고충실도 시뮬레이터를 이용하여 실습하였다. 9개의 조가 동시에 실습에 참여할 수 있는 여건이 아닌 관계로 실습실과 대기실로 나누어 진행하였다. 1개의 조가 먼저 15분씩 연습을 하고 다시 교차하여 15분씩 연습하도록 하였으며, 교수-학생 상호작용 과정에서 환자에 대한 추가정보를 제공하였다. 나머지 학생들은 산부 간호에 대한 시나리오 사정 및 팀 기반 학습, 간호사와 대상자 역할극 등을 통해 자유롭게 시뮬레이션 상황에 대한 이해와 실기 훈련을 시행하였다. 4주차 세션은 고충실도 시뮬레이터를 이용하여 실습을 진행하는 단계로 한 조당 소요되는 시간은 약 15분이었으며, 4명의 학생들은 분만실 간호사 3명, 보호자 1명으로 역할을 구분하여 진행하였다. 간호사 역할은 대상자를 관찰하여 상황을 보고하는 간호사 1인, 보고 받은 내용을 전달받고 기록을 담당하는 간호사 1인, 대상자에게 중재를 시행하고 보호자를 지지하는 간호사 1인으로 나누어 진행하였다. 훈련하는 동안 연구 보조원은 필요 시 의사 역할을 하며 학생들의 시뮬레이션 운영을 도왔다. 5주차 디브리핑 세션은 교수자와 모든 학생들이 한 장소에 모여 각자의 경험 및 강점과 약점에 대한 발표와 토론, 시뮬레이션 상황에 대한 정리와 학생들의 대처에 대한 분석과 조언 등으로 정리 및 반영하는 시간을 가졌다(Table 1).

• 대조군: 본 연구의 대조군의 교육프로그램 중재 방법으로 분만 시연 실습 모형을 이용한 분만 간호 중재를 제공하였다. 교육프로그램 1주차 세션은 실험군과 동일하게 산부 간호 교육 및 핵심 이론에 대하여 2시간 동안 설명하였다. 2–3주 세션에서는 분만 관련 핵심 간호술기에 대한 훈련을 시행하는 단계로 36명이 각각 4명씩 한 조를 이루어 총 9조로 구성되어 실습에 참여하였다. 산부 간호에 대한 개별 및 팀 기반 학습을 통해 분만 관련 핵심 간호술기를 수행할 수 있도록 하였다. 분만 시연 실습 모형을 이용하였으며, 훈련하는 동안 연구 보조원은 학생들의 핵심 간호술기 훈련을 도왔다. 9개의 조가 동시에 실습에 참여할 수 있는 여건이 아닌 관계로 두 군데로 나누어 각각 핵심 간호술기를 연습하도록 하였으며, 2개의 조가 먼저 15분씩 연습을 하고 다시 교차하여 15분씩 연습하도록 하였다. 나머지 학생들은 산부 간호에 대한 개별 및 팀 기반 학습을 하도록 하면서 정해진 시간에 술기 연습을 진행하도록 구성하였다. 4주차 세션에서는 주제와 관련된 산부 간호에 대하여 자료를 수집하고 간호문제를 파악하여 간호계획을 세우도록 하였다. 평가는 각 조별로 한 장소에 모여 개별 및 조별로 정상분만 과정 및 산부 간호에 대하여 발표하였으며, 교수자 1인은 발표 내용에 대한 분석과 평가를 하였다(Table 1).

#### 사후 조사

사후 조사는 두 군 모두 5주차에 분만 간호 지식, 비판적 사고성향, 임상수행능력에 대해 설문지로 조사하였으며, 작성시간은 15분 정도가 소요되었다(Table 1). 설문지 응답 내용은 무기명으로 처리되었고 대상자가 설문지를 작성하는 동안 연구자로부터 영향을 받지 않도록 자리를 비워 두었다. 또한, 시뮬레이션 교육프로그램이 모두 종료된 후 대조군에게도 실험군과 동일한 교육을 제공하였으며, 설문조사가 완료된 후에는 감사의 표시로 소정의 상품을 제공하였다.

### 자료 분석

본 연구에서 수집된 자료는 IBM SPSS for Windows ver. 24.0 (IBM Corp., Armonk, NY, USA)을 이용하여 다음과 같이 분석하였다.

1) 대상자의 일반적 특성은 빈도, 백분율, 평균, 표준편차로 산출하였다.

2) 실험군과 대조군의 일반적 특성에 따른 동질성 검정은 chi-square test 또는 Fisher exact test와 independent t-test로 분석하였다.

3) 실험군과 대조군의 분만 간호 지식, 비판적 사고성향, 임상수행능력은 independent t-test로 분석하였으며, 본 연구의 가설에 따라 유의수준(α)=.05, 단측 검정으로 분석하고 *p*<.025면 유의성이 있다고 설정하였다[27].

## Results

### 대상자의 특성 및 동질성 검정

본 연구 대상자는 고충실도 시뮬레이터를 이용한 분만 간호 교육에 참여한 간호학과 3학년 학생 72명으로, 대조군 간 일반적 특성과 사전 간호 지식, 비판적 사고성향, 임상수행능력에 대한 종속변수의 동질성 검증 결과 통계적으로 유의한 차이가 없어 모두 동질한 것으로 나타났다(Table 2). 평균 연령은 실험군 23.11±1.98세, 대조군 22.61±1.74세였으며, 두 집단 간 유의한 차이가 없었다. 실험군의 여학생이 88.9% (32명), 남학생이 11.1% (4명)였고, 대조군의 여학생이 86.1% (31명), 남학생이 13.9% (5명)로 두 집단 간 유의한 차이가 없었다. 의사표현 점수는 실험군 2.47점, 대조군 2.58점으로 나타났고, 간호학과 지원 동기로는 취업률 고려가 가장 높았으며 실험군 63.9% (23명), 대조군 44.4% (16명)로 두 집단 간 유의한 차이가 없었다. 전공 만족도 점수는 실험군 2.63, 대조군은 2.55로 두 집단 간 유의한 차이가 없었다. 대인관계 정도는 실험군 2.25점, 대조군 2.41점으로 나타났고, 병원실습 중 가장 어려운 점은 두 군 모두 과제라고 응답하였으며 실험군 50.0% (18명), 대조군 52.8% (19명)로 두 집단 간 유의한 차이가 없었다. 선호하는 수업 방식은 실험군은 강의식 수업 41.7% (15명), 사례기반 학습 25.0% (9명), 대조군은 강의식 수업 52.8% (19명), 실습 위주 학습법 13.9% (5명) 순으로 나타났으며, 두 집단 간 유의한 차이가 없었다. 전 학기 여성건강간호학 성적은 실험군 2.22점, 대조군 2.63점으로, 두 집단 간 유의한 차이가 없었다.

분만 간호 지식은 실험군 13.19점/대조군 13.63점(t=–1.26, *p*=.105), 비판적 사고성향은 실험군 95.11점/대조군 95.61 (t=–0.25, *p*=.488), 임상수행능력은 실험군 156.41점/대조군 159.72점(t=–0.60, *p*=.309)으로 유의한 차이가 없어 종속변수 모두 동질한 것으로 나타났다(Table 2).

### 가설 검정

고충실도 시뮬레이터를 이용한 분만 간호 교육프로그램이 분만 간호 지식, 비판적 사고성향, 임상수행능력에 미치는 효과를 확인하기 위한 가설 검정 결과는 다음과 같다(Table 3).

#### 제1 가설

가설 검정 결과, 실험군의 사후 분만 간호 지식 평균은 14.41±0.90점으로 대조군의 13.86±1.09점보다 통계적으로 유의하게 높게 나타나(t=2.33, *p*=.011) 제1 가설은 지지되었다.

#### 제2 가설

가설 검정 결과, 실험군의 사후 비판적 사고성향 평균은 106.05±9.88점으로 대조군의 98.77±6.23점보다 통계적으로 유의하게 높게 나타나(t=3.73, *p*<.001) 제2 가설은 지지되었다.

#### 제3 가설

가설 검정 결과, 실험군의 사후 임상수행능력 평균은 182.72±20.75점으로 대조군의 170.38±20.50점보다 통계적으로 유의하게 높게 나타나(t=2.53, *p*=.006) 제3 가설은 지지되었다.

## Discussion

본 연구는 간호대학생 대상의 고충실도 시뮬레이터를 이용한 분만 간호 교육프로그램을 적용하여 분만 간호 지식, 비판적 사고성향, 임상수행능력에 미치는 효과를 평가하고자 시도하였다.

본 연구에서 고충실도 시뮬레이터를 이용한 분만 간호 교육프로그램을 받은 실험군은 대조군보다 분만 간호 지식이 향상되었다. 이러한 결과는 간호대학생을 대상으로 문제중심 학습 통합 시뮬레이션 교육프로그램이 간호 지식을 향상시켰음을 보고한 선행연구[28] 결과와 유사하였다. 시뮬레이션을 이용한 교육은 학생들의 지식을 향상시킨다는 결과가 여러 연구를 통해 보고된 바 있다[14,28]. 실제 출산상황과 유사한 시나리오를 시뮬레이션 실습에 적용하고 직접 간호술기를 수행하는 과정에서 즉각적으로 피드백이 이루어졌기 때문이라 생각된다.

비판적 사고성향은 고충실도 시뮬레이터를 이용한 분만 간호 교육프로그램을 받은 실험군이 대조군에 비해 통계적으로 유의하게 향상되었다. 이는 간호대학생 대상으로 시뮬레이션 교육을 적용하여 비판적 사고능력이 향상되었다고 보고한 선행연구[28]와 유사한 결과이며, Simmom을 이용한 산후 출혈 산모 시뮬레이션 교육을 적용 후 실험군의 비판적 사고성향 점수가 대조군보다 증가하였으나 통계적으로 유의한 차이가 없었다고 보고한 선행연구[7]와는 차이를 보였다. 비판적 사고는 어떤 문제 상황에서 문제 해결을 위해 적용할 수 있는 충분한 지식이 있어야 하며 지속적인 학습을 통해 다양한 상황에 대한 접근이 필요하다[21]. 따라서 본 연구에서는 분만을 위해 입원 중인 임신 39주 2일의 초산부가 정상 질식분만 상황에서 자궁 수축 및 태아 심박동 확인을 경험하는 과정을 포함함으로써 학생들이 자궁 수축과 관련된 사정 요소와 복부 촉진법, 분만 시 안녕 상태 파악 및 태아 심박동 조절과 관련된 태아 산소공급 문제 등에 대한 학습이 가능하였으며, 비판적 사고능력을 향상시킬 수 있었을 것이라 생각된다.

임상수행능력은 고충실도 시뮬레이터를 이용한 분만 간호 교육프로그램을 받은 실험군이 대조군에 비해 통계적으로 유의하게 향상되었다. 이러한 결과는 간호대학생을 위한 시뮬레이션 교육프로그램의 효과를 검증한 선행연구[7,29,30]의 결과와 유사하였다. 이는 시뮬레이션 기반 교육이 대상자로 하여금 사전 학습을 통해 이론적 지식을 습득하게 하고 임상경험이 전혀 없는 상태에서 임상과 유사한 상황에 노출되면서 문제 해결방식을 학습하게 함으로써 임상수행능력 향상에 도움을 주었을 것이라 생각된다. 이상의 논의를 토대로 본 연구에서 개발된 고충실도 시뮬레이터를 이용한 분만 간호 교육은 간호대학생들의 분만간호 지식, 비판적 사고성향 및 임상수행능력을 향상시킴을 알 수 있었다. 간호대학생들은 고충실도 시뮬레이터를 이용한 분만 간호 교육프로그램을 통해 분만 간호에 대한 새로운 지식과 경험을 얻는 기회가 되었다. 더욱이 본 연구에서 개발된 사례를 적용한 고충실도 시뮬레이션 학습은 실제 상황과 같이 현장감이 있고 상황에 몰입할 수 있어 분만 간호에 대해 긍정적인 교육 효과를 확인할 수 있었다.

본 연구는 실험군과 대조군을 분반하여 다른 층에 배정하였으나, 같은 학년의 학생들을 대상으로 같은 시기에 중재를 함으로써 실험의 확산 위험을 완전히 배제하지 못한 제한점이 있다. 따라서 연구 결과의 확대 해석이나 일반화에 신중을 기해야 할 것이다. 추후 내적타당도에 위협을 줄 수 있는 외생변수가 배제된 효과적인 연구 설계를 하여 시뮬레이션 실습교육의 효과를 확인하는 연구가 필요하다.

이러한 제한점에도 불구하고, 본 연구의 대상자는 3학년 1학기 모성간호학 전공과목을 이수하였으며, 시뮬레이션 및 임상실습 경험이 없었음을 고려하여 볼 때 임상현장과 유사한 고충실도 시뮬레이터를 활용하여 사전 전공지식의 효과를 확인하였다는 점이 본 연구의 의의가 될 수 있다. 향후 시뮬레이션 교육 적용 시 대상자의 임상실습에 대한 요구도 파악 및 분석을 통해 시뮬레이션 기반 교육의 내용을 구성하여 사전에 교육함으로써 시뮬레이션 교육의 성과를 극대화할 수 있을 것이다. 이를 바탕으로 간호대학생에게 간호학적 지식과 실제 임상현장 사례를 반영한 시뮬레이션 교육을 적용한다면 임상에서 요구하는 핵심능력 향상에 기여할 수 있을 것으로 생각된다. 또한 간호대학생들의 임상 실무에 대한 문제해결 역량을 향상시키기 위해서는 여성 건강과 관련한 다양하고 구체적인 임상사례들을 개발하고 이를 시뮬레이션 교육과 접목하는 체계적인 교수 학습전략이 필요할 것이다. 즉 실제 임상현장의 상황을 기반으로 유사한 시뮬레이션 실습 환경을 제공하는 것은 지금까지 임상실습을 통해서 도달할 수 있었던 다양한 실무능력을 향상시키는 데 도움이 될 것이다.

## Figures and Tables

**Table 1.** Research procedures of the two groups

Session	Experimental group	Control group
1	• Pre-test: General characteristics, nursing knowledge, critical thinking, clinical performance (20 minutes)	
	• Orientation & prerequisite learning (120 minutes)	
2–3	• Scenario assessment (20 minutes)	• Individual learning (40 minutes)
	- Case study scenario: first stage of labor	• Team learning (40 minutes)
	- General physical assessment	• Core skills practice using a delivery model (40 minutes)
	- Leopold’s maneuver	- Nursing skills related to the stage of labor
	- NST monitoring (checking the fetal heart rate)	
	- Measuring the height of the fundus	
	- Education on breathing technique management	
	- Report on client’s condition	
	- Nursing interventions for anxiety and pain	
	- Administering oxytocin	
	• Intervention training (40 minutes)	
	• Team learning and role play (30 minutes)	
	• Core skills using HFS (SimMom) (30 minutes)	
	- Nursing skills related to the scenario	
4	• Simulation running using HFS (SimMom) (9 teams/120 minutes)	• Scenario assessment (30 minutes)
		- Case study scenario: first stage of labor
		- Team and individual learning
		- Explanation for nursing diagnosis and plan related to labor stages
		• Recording worksheet (30 minutes)
		• Feedback and evaluation (60 minutes)
5 (Post-test)	• Debriefing and evaluation (100 minutes)	
	- Post-test: Nursing knowledge, critical thinking, clinical performance (15 minutes)	

HFS: High fidelity simulator, NST: non-stress test. SimMom (Laerdal, Stavanger, Norway).

**Table 2.** Homogeneity test of characteristics and variables between the experimental and control groups (N=72)

Characteristic	Categories		n (%)		χ²/t	*p*
			Experimental (n=36)	Control (n=36)		
Age (year)		Mean±SD	23.11±1.98	22.61±1.74	1.13	.709
Gender	Man		4 (11.1)	5 (13.9)	.000	1.00
	Woman		32 (88.9)	31 (86.1)		
Level of self-expression		Mean±SD	2.47±0.77	2.58±0.87	–0.57	.378
Motivation for nursing	Considerations of employment		23 (63.9)	16 (44.4)		.175^†^
	Recommendation of others		3 (8.3)	8 (22.2)		
	Considerations of aptitude		9 (25.0)	10 (27.8)		
	Considerations of grades		1 (2.8)	2 (5.6)		
Satisfaction with nursing department		Mean±SD	2.47±0.61	2.61±0.68	–0.91	.748
Satisfaction with clinical practice		Mean±SD	2.63±0.72	2.55±0.87	0.44	.182
Human relationships		Mean±SD	2.25±0.64	2.41±0.81	–0.96	.112
Most difficult part of clinical practice	Human relationships		11 (30.3)	12 (33.3)	1.30	.897
	Differences between disciplines		7 (19.4)	5 (13.9)		
	Assignments		18 (50.0)	19 (52.8)		
Favorite	Lectures		15 (41.7)	19 (52.8)		.339^†^
learning method	Problem-based learning		4 (11.1)	4 (11.1)		
	Discussion-based learning		1 (2.8)	4 (11.1)		
	Clinical practice		7 (19.4)	5 (13.9)		
	Case-based learning		9 (25.0)	4 (11.1)		
Grade in women’s health nursing		Mean±SD	2.22±0.89	2.63±1.15		
	High		9 (25.0)	7 (19.4)		.355^†^
	Moderate		25 (69.4)	23 (63.9)		
	Low		2 (5.6)	6 (16.7)		
Nursing knowledge		Mean±SD	13.19±2.02	13.63±0.59	–1.26	.105
Critical thinking disposition		Mean±SD	95.11±7.84	95.61±8.49	–0.25	.488
Clinical performance		Mean±SD	156.41±22.87	159.72±23.85	–0.60	.309

† Fisher exact test.

**Table 3.** Comparisons of nursing knowledge, critical thinking disposition, and clinical performance scores between experimental and control groups after the intervention (N=72)

Variable	Mean±SD		t	*p*
	Experimental (n=36)	Control (n=36)		
Nursing knowledge	14.41±0.90	13.86±1.09	2.33	.011
Critical thinking disposition	106.05±9.88	98.77±6.23	3.73	<.001
Clinical performance	182.72±20.75	170.38±20.50	2.53	.006
